# Supplementary material for: Origin of the Rich Polymorphism of Gold in Penta-Twinned Nanoparticles
Source: Nano Lett. 2025 Feb 18;25(9):3588–96. doi: 10.1021/acs.nanolett.4c06473 (PMC11887444; doi:10.1021/acs.nanolett.4c06473)
Supplement: Supplementary file 1 — nl4c06473_si_001.pdf [file nl4c06473_si_001.pdf]

Supporting Information

Origin of the rich polymorphism of gold in penta-twinned nanoparticles

Camino Martín-Sánchez[a,b*], Ana Sánchez-Iglesias[c], José Antonio Barreda-Argüeso[b], Jean-Paul Itié[d], Paul Chauvigne[d], Luis M. Liz-Marzán[e,f], Fernando Rodríguez[b]

[a] Faculté des Sciences, Département de Chimie Physique, Université de Genève, 30 Quai Ernest-Ansermet, CH-1211 Genève, Switzerland
[b] MALTA Consolider, DCITIMAC, Facultad de Ciencias, University of Cantabria, Av. Los Castros 48, Santander, 39005, Spain
[c] Centro de Física de Materiales (CSIC-UPV/EHU), Paseo Manuel de Lardizabal 5, 20018 Donostia-San Sebastián, 20118, Spain
[d] Synchrotron SOLEIL, L'Orme des Merisiers St.Aubin, BP48, 91192 Gif-sur-Yvette, France
[e] CIC biomaGUNE, Basque Research and Technology Alliance (BRTA), Paseo de Miramón 194, Donostia-San Sebastián, 20014, Spain
[f] Ikerbasque, Basque Foundation for Science, Bilbao, 43018, Spain

1) Single crystal and penta-twinned gold nanorods. Relevant crystallographic directions and mismatch gap of penta-twinned nanoparticles

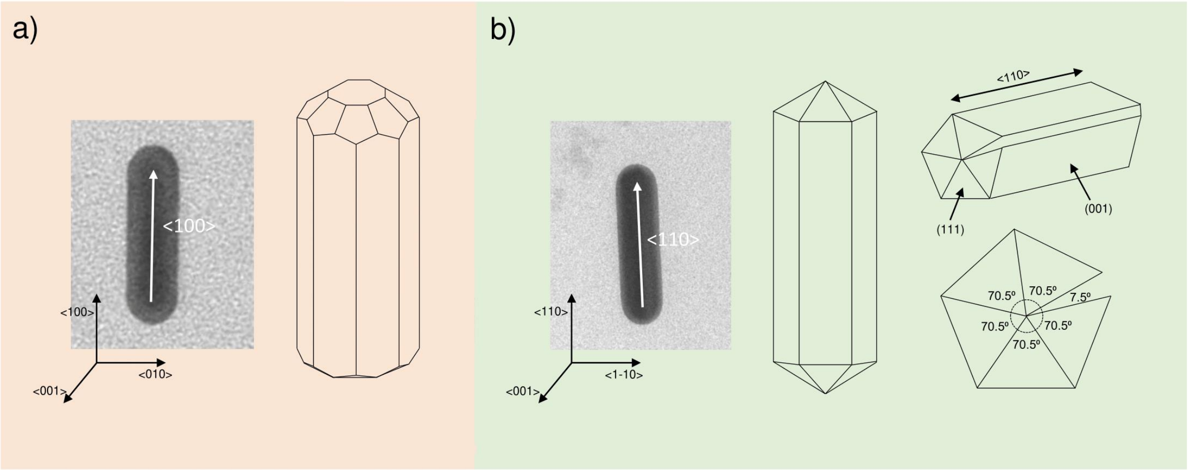

Figure S1. TEM images of single crystal a) and penta-twinned b) gold nanorods and schematic representation of their habits in terms of crystallographic planes. The crystallographic directions along the long axis and the two perpendicular directions are indicated. The 7.5º mismatch angle created by the five *fcc* single crystals forming the penta-twinned nanoparticle is indicated.

2) Experimental details

Nanoparticle synthesis:

Chemicals: Gold (III) chloride trihydrate ($HAuCl_4$, ≥ 99 %), hexadecyltrimethylammonium bromide (CTAB, ≥ 99 %), hexadecyltrimethylammonium chloride (CTAC, 25 wt % in water), benzyldimethylhexadecylammonium chloride (BDAC, 99 %), citric acid (≥ 99.5 %), sodium borohydride ($NaBH_4$, ≥ 98 %), silver nitrate ($AgNO_3$, ≥ 99 %), hydrochloric acid (HCl, 37 %) and L-ascorbic acid (AA, ≥ 99%) were purchased from Sigma-Aldrich. α-Methoxy-ω-mercapto poly (ethylene glycol), PEG-SH, Mw: 5 K) was purchased from Rapp Polymere. Ethanol (99.9 %) and methanol (99.9 %) were purchased from Scharlab. All reactants were used without further purification. Milli-Q water (resistivity 18.2 MΩ·cm at 25 °C) was used in all experiments. All glassware magnetic bars were washed with aqua regia, rinsed with Milli-Q water, and dried before use.

Synthesis of gold seeds [1-3]: To an aqueous solution of CTAC (10 mL, 50 mM) was added a solution of $HAuCl_4$ (0.05 mL, 50 mM) followed by stirring for ~3 minutes until complexation of gold precursor with surfactant (transparent, yellowish solution). Next, citric acid (0.05 mL, 1 M) was added followed by the injection of freshly prepared $NaBH_4$ (0.25 mL, 25 mM) under vigorous stirring at room temperature. The mixture turned from light yellow to brownish indicating the formation of gold seeds. After two minutes, the vial was closed and the seed solution was heated in an oil bath at 80 ºC during 90 minutes under gently stirring, to induce twin formation. The aging process led to a gradual color change from brown to red. The thermally treated seed solution (concentration of metallic gold 0.25 mM) was removed from the oil bath and stored at room temperature and was used for the synthesis of nanorods, bipyramids and gold decahedra.

Synthesis of penta-twinned gold nanorods: To prepare the growth solution, 75 mg of CTAB was dissolved in an aqueous CTAC solution (100 mL, 100 mM). The mixture was kept under stirring at 30 °C for 10 min in a water bath. After dissolution of the binary surfactant mixture, $HAuCl_4$ solution (0.5 mL, 25 mM) was added under stirring at 30 ºC to obtain a transparent yellowish solution and the mixed solution was kept at 40 ºC for 15 min. Subsequently, AA solution (0.188 mL, 100 mM) was added under stirring and the colour of the growth solution changed from yellowish to colorless. Finally, gold seed solution (0.376 mL, 0.25 mM) was injected into the growth solution under vigorous stirring for 30 s. The mixture was then kept undisturbed at 40 ºC for 1h in the water bath. The solution was centrifuged twice (3900 RCF, 30 min) to remove excess of reagents, and redispersed in CTAB 1 mM to a final gold concentration of 1mM. The final dimensions of gold nanorods were 55 ± 2 nm (length) and 24 ± 1 nm (width).

Synthesis of gold bipyramids: To synthesize gold bipyramids, an aliquot of gold seeds (1.88 mL) was added under vigorous stirring to an aqueous growth solution of CTAB (100 mL, 100 mM), $HAuCl_4$ (5 mL, 10 mM), $AgNO_3$ (1 mL, 10 mM), HCl (2 mL, 1M) and AA (0.8 mL, 100 mM). The mixture was left undisturbed at 30 °C for 2 hours. The solution containing gold bipyramids was centrifuged twice (3900 RCF, 30 min) to remove excess of reagents, and redispersed in CTAB 1 mM to a final gold concentration of 1mM. The final dimensions of gold bipyramids were 71 ± 3 nm (length) and 19 ± 1 nm (width).

Synthesis of gold decahedra: A given volume of gold seeds (0.36 mL and 0.1 mL for 31 nm and 49 nm decahedra, respectively) was added under vigorous stirring to an aqueous growth solution

of BDAC (50 mL, 100 mM), $HAuCl_4$ (0.5 mL, 50 mM) and AA (0.375 mL, 100 mM) at 30 ºC. The mixture was gently stirring at 30 °C for 30 minutes. The solution was centrifuged twice (6940 RCF and 5310 RCF for 31 nm and 49 nm, respectively, 30 min) to remove excess of reagents, and redispersed in CTAB 1 mM. The final concentration of gold was 1 mM. The final dimensions of gold decahedra were 31 ± 1 nm and 49 ± 1 nm (edge length).

Ligand exchange [4]: To replace the cationic surfactant (CTAB) and transfer the gold nanoparticles (nanorods, bipyramids, and decahedra) to alcoholic mixture (MeOH-EtOH, 4:1, in volume), α-methoxy-ω-mercapto poly (ethylene glycol) (PEG-SH, Mw: 5K) was used. A freshly prepared aqueous solution of PEG-SH (32 mg, for gold nanorods and gold bipyramids, and 25 mg and 20 mg for gold decahedra of 31 nm and 49 nm, respectively, dissolved in 2 mL of water), was added dropwise under stirring to a dispersion of gold nanoparticles (10 mL, 1 mM) in CTAB 1 mM. The solution was left for 2 h under gently stirring, and then centrifuged twice (using the same conditions as above) in methanol-ethanol 4:1. Pegylated gold nanoparticles (nanorods, bipyramids, and decahedra) were finally dispersed in the alcoholic mixture solution to achieve an optical density (1 cm of path length) of around 70 for all samples. The final gold concentrations for the different shapes were 8.7 mM (nanorods), 3.5 mM (bipyramids), 10.5 mM and 8.0 mM for gold decahedra of 31 nm and 49 nm, respectively

Instrumentation: Transmission electron microscopy (TEM) images were acquired on a FEI Tecnai G2 20 TWIN microscope operating at an acceleration of 200 kV. Optical extinction spectra were recorded using an Agilent Cary 3500 UV-visible spectrophotometer. The dimensions of the gold nanoparticles were determined by TEM analysis by measuring more than 200 nanoparticles per sample randomly chosen. The gold concentration was determined by the absorbance at 400 nm, which is assumed to have a size and shape-independent absorption coefficient [5].

Inductively coupled plasma mass spectrometry (ICP-MS): ICP-MS measurements were conducted on gold bipyramids for Au and Ag content determination which yielded a composition of 3 wt % Ag (97 wt % Au). Gold bipyramids were digested in freshly prepared aqua regia (HNO_3:HCl, 3:1, v/v) at room temperature for 2 h, diluted water, and then subjected to ICP-MS analysis. ICP-MS measurements were performed on an iCAP-Q (ThermoFisher Scientific) equipped with an automatic injector ASX-520 (CETAC Technologies Inc.).

X-ray diffraction measurements:

XRD measurements on PT-AuNP MeOH-EtOH 4:1 colloids were performed at the SOLEIL Synchrotron (France) using the PSICHÉ beamline. PT-AuNP colloidal dispersions were measured in a diamond anvil cell (DAC) into a 150 μm diameter hole within a rhenium gasket preindented to a thickness of 35 μm. Compacted gold powder of 2 μm average grain size was also loaded to precisely compare lattice parameters between systems under the same experimental conditions. We used a DAC because the PSICHÉ beamline is designed to perform angle-dispersive powder XRD using a DAC as a sample holder. Additionally, the DAC provides a reliable seal for the sample, ensuring optimal conditions for the XRD measurements. Pressure within the DAC was precisely controlled within 0.05 GPa using micro gold powder Bragg peaks [6]. This resulted in highly accurate zero-pressure measurements, with a relative volume change

($\Delta V/V$) uncertainty of less than 0.03%, suitable for precise lattice parameter determinations. A parallel configuration geometry for diffraction (incident X-ray beam parallel to the DAC load axis) was used. 2D XRD data were collected on a CdTe2M Dectris detector using a monochromatic X-ray beam with a wavelength of 0.3738 Å, focused to a beam size of 12×14 μm^2 (FWHM). The 2D XRD patterns were treated with the Dioptas program [7], and the intensity $I(2\theta)$ patterns were analyzed using the Match! Software [8]. LeBail fits were accomplished using Gaussian line profiles using two body-centered cells with a tetragonal *I4/mmm* space group for AuDec and an orthorhombic *Immm* space group for AuBip and AuRod. Instrumental resolution parameters were determined using a sample of CeO_2 with high crystalline quality using the Caglioti equation [9]: U = -0.009421, V = -0.008602 y W = 0.002219, to account for the instrumental broadening: $B_{inst}^2 = \mathrm{W} + \mathrm{V} \tan\theta + \mathrm{U} \tan^2\theta$. Subsequently, U, V and W were kept constant throughout the analysis. The lattice parameters were determined with an accuracy better than 0.003 Å, and the FWHM of the Bragg peaks, B_r, were determined with a precision of 0.001° using the equation $B_{exp}^2 = B_{inst}^2 + B_r^2$.

3) Lattice correspondence between *fcc* and *bct*

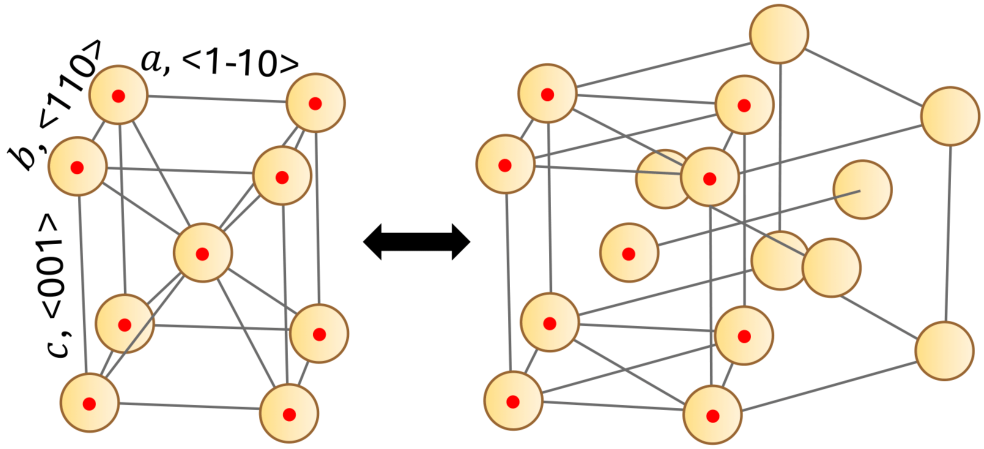

Figure S2. Schematic representation of the relation between body-centered tetragonal (left) and face-centered cubic (right) cells.

4) Elastic model for PT-AuNP

If we consider a stress along the <001> axis, the strain along the <001> and <1-10> directions – perpendicular to the <110> twin axis- in each single-crystal portion is given by:

$$\varepsilon_{1-10} = \varepsilon_l = \frac{\Delta l}{l} = \frac{a'}{a}\frac{\sin 36}{\sin 35.25} - 1 \tag{1}$$

$$\varepsilon_{001} = \varepsilon_h = \frac{\Delta h}{h} = \frac{a'}{a}\frac{\cos 36}{\cos 35.25} - 1 \tag{2}$$

Where a and a' are the sides of the unstressed and stressed single-crystal domains, respectively, given by:

$$a = \sqrt{h^2 + \left(\frac{l}{2}\right)^2} \tag{3}$$

$$a' = \sqrt{h^2(1+\varepsilon_{001})^2 + \left(\frac{l}{2}\right)^2 (1+\varepsilon_{1-10})^2} \approx a\left[1 + \left(\frac{h}{a}\right)^2 \varepsilon_{001} + \left(\frac{l}{2a}\right)^2 \varepsilon_{1-10}\right] \tag{4}$$

Therefore, combining Eqs. (1) and (2) with Eqs. (3) and (4), we finally obtain the strains along the (110) plane:

$$\varepsilon_{1-10} = [1 + \cos^2 35.25\, \varepsilon_{001} + \sin^2 35.25\, \varepsilon_{1-10}]\frac{\sin \alpha}{\sin 35.25} - 1 \tag{5}$$

$$\varepsilon_{001} = [1 + \cos^2 35.25\, \varepsilon_{001} + \sin^2 35.25\, \varepsilon_{1-10}]\frac{\cos \alpha}{\cos 35.25} - 1 \tag{6}$$

Additionally, the strain-stress tensor for a cubic system referred to an arbitrary orthogonal coordinate axis set (x,y,z) is given by [10]:

$$\begin{bmatrix} \varepsilon_{xx} \\ \varepsilon_{yy} \\ \varepsilon_{zz} \\ \varepsilon_{yz} \\ \varepsilon_{zx} \\ \varepsilon_{xy} \end{bmatrix} = \begin{bmatrix} \frac{1}{E_x} & -\frac{\upsilon_{yx}}{E_y} & -\frac{\upsilon_{zx}}{E_z} & & & \\ -\frac{\upsilon_{xy}}{x} & \frac{1}{E_y} & -\frac{\upsilon_{zy}}{E_z} & & 0 & \\ -\frac{\upsilon_{xz}}{E_x} & -\frac{\upsilon_{yz}}{E_y} & \frac{1}{E_z} & & & \\ & & & \frac{1}{G_{yz}} & & \\ & 0 & & & \frac{1}{G_{zx}} & \\ & & & & & \frac{1}{G_{xy}} \end{bmatrix} \begin{bmatrix} \sigma_{xx} \\ \sigma_{yy} \\ \sigma_{zz} \\ \sigma_{yz} \\ \sigma_{zx} \\ \sigma_{xy} \end{bmatrix} \tag{7}$$

where ε refers to strain, σ is the stress, E is the Young's modulus, υ is the Poisson's ration, and G represents the shear modulus in the axes of interest (x, y, z). Additionally, the Young's modulus and the Poisson's ratio for cubic crystals are given by the expressions:

$$\frac{1}{E_{hkl}} = S_{11} - 2\left[(S_{11} - S_{12}) - \frac{1}{2}S_{44}\right](m^2n^2 + n^2p^2 + m^2p^2) \tag{8}$$

$$\upsilon_{\alpha\beta} = \frac{S_{12} + \left(S_{11} - S_{12} - \frac{1}{2}S_{44}\right)\left(m_\alpha^2 m_\beta^2 + n_\alpha^2 n_\beta^2 + p_\alpha^2 p_\beta^2\right)}{S_{12} - 2\left(S_{11} - S_{12} - \frac{1}{2}S_{44}\right)\left(m_\alpha^2 n_\alpha^2 + n_\alpha^2 p_\alpha^2 + m_\alpha^2 p_\beta^2\right)} \tag{9}$$

where S_{ij} are the single crystal elastic compliances, the terms m, n, and p are the direction cosines: the cosine of the angle between the direction of interest <hkl> and the X-, Y-, and Z-axes (the <100> directions). α and β are the stress and the in-plane directions, respectively. For gold, considering the elastic constants reported elsewhere [11] using Eqs. (8) and (9), the strain-stress tensor results in:

$$\begin{bmatrix} \sigma_{(110)} \\ \sigma_{(1-10)} \\ \sigma_{(001)} \\ \sigma_{(1-10)(001)} \\ \sigma_{(110)(001)} \\ \sigma_{(110)(0-10)} \end{bmatrix} = \begin{bmatrix} \sigma_{xx} \\ \sigma_{yy} \\ \sigma_{zz} \\ \sigma_{yz} \\ \sigma_{zx} \\ \sigma_{xy} \end{bmatrix} = \begin{bmatrix} 213.2 & 136.3 & 160.5 & & & \\ 136.3 & 213.2 & 160.5 & & 0 & \\ 161.2 & 161.2 & 187.5 & & & \\ & & & 0.89 & & \\ & 0 & & & 0.46 & \\ & & & & & 0.35 \end{bmatrix} \begin{bmatrix} \varepsilon_{xx} \\ \varepsilon_{yy} \\ \varepsilon_{zz} \\ \varepsilon_{yz} \\ \varepsilon_{zx} \\ \varepsilon_{xy} \end{bmatrix} \tag{10}$$

Consequently, for the <110>, <1-10>, and <001> directions we have:

$$\sigma_{110} = 213.2\varepsilon_{110} + 136.3\varepsilon_{1-10} + 160.5\varepsilon_{001} \tag{11}$$

$$\sigma_{1-10} = 136.3\varepsilon_{110} + 213.2\varepsilon_{1-10} + 160.5\varepsilon_{001} \tag{12}$$

$$\sigma_{001} = 161.2\varepsilon_{110} + 161.2\varepsilon_{1-10} + 187.5\varepsilon_{001} \tag{13}$$

References

[1] Sánchez-Iglesias, A.; Winckelmans, N.; Altantzis, T.; Bals, S.; Grzelczak, M.; Liz-Marzán, L. M. High-Yield Seeded Growth of Monodisperse Pentatwinned Gold Nanoparticles through Thermally Induced Seed Twinning. *J. Am. Chem. Soc*. **2017**, 139, 107-110.

[2] Sánchez-Iglesias, A.; Grzelczak, M. Expanding Chemical Space in the Synthesis of Gold Bipyramids. *Small* **2025**, 21, 2407735.

[3] Sánchez-Iglesias, A.; Jenkinson, K.; Bals, S.; Liz-Marzán, L. M.. Kinetic Regulation of the Synthesis of Pentatwinned Gold Nanorods below Room Temperature. *J. Phys. Chem. C* **2021**, 125, 23937-23944.

[4] García-Álvarez, R.; Hadjidemetriou, M.; Sánchez-Iglesias, A.; Liz-Marzán, L. M.; Kostarelos, K. In vivo Formation of Protein Corona on Gold Nanoparticles. The Effect of their Size and Shape. *Nanoscale* **2018**,10, 1256-1264.

[5] Rodríguez-Fernández, J.; Pérez-Juste, J.; Mulvaney, P.; Liz-Marzán, L. M. Spatially-Directed Oxidation of Gold Nanoparticles by Au (III)− CTAB Complexes. *J. Phys. Chem. B* **2005**, 109, 14257-14261.

[6] Heinz, D. L.; Jeanloz, R. The Equation of State of the Gold Calibration Standard. *J. Appl. Phys*. **1984**, 55, 885– 893.

[7] Prescher, C.; Parakapenka, V. B. DIOPTAS: a Program for Reduction of Two-Dimensional X-Ray Diffraction Data and Data Exploration. *High Press. Res.* **2015**, 35, 223–230

[8] Match! - Phase Analysis using Powder Diffraction, Crystal Impact - Dr. H. Putz & Dr. K. Brandenburg GbR, Kreuzherrenstr. 102, 53227 Bonn, Germany.

[9] Caglioti, A.; Paoletti, A.; Ricci, F. P.; Choice of Collimators for a Crystal Spectrometer for Neutron Diffraction. *Nucl. Instr.* **1958**, 3, 223-228.

[10] Hopcroft, M. A.; Nix, W. D.; Kenny, T. W. What is the Young's Modulus of Silicon? *J. Microelectromechanical Syst.* **2010**, 19, 229-238.

[11] Martienssen, W. *Springer Handbook of Condensed Matter and Materials Data*; Springer Science & Business Media, 2006.
